# Protein‐enriched fiber *vegan meal* promote satiety and suppress food intake in rats

**DOI:** 10.1002/fsn3.1570

**Published:** 2020-04-14

**Authors:** Xue Wang, Qi‐Le Zhou, Yi Yang, Yan‐Li Wei, Wei Wei, Hong‐Xia Bi, Dong Li, Shi‐Long Jiang

**Affiliations:** ^1^ Heilongjiang Feihe Dairy Co., Ltd. Beijing China; ^2^ Beijing Institute of Nutritional Resources Beijing Academy of Science and Technology Beijing China

**Keywords:** food intake, potato protease inhibitor II, protein‐enriched vegan meal, satiation signals, satiety

## Abstract

Dietary preferences were closely associated with the pathogenesis of numbers of metabolic disorders, in particularly, obesity. Dietary fiber was shown to be capable of preventing weight gain and excessive food intake mainly through stimulating chewing and saliva secretion, and promote satiety signals. In this study, we characterized the "Vitamin World^®^ Vegan Meal" Formula of *Feihe*, a novel protein‐enriched fiber dietary supplement contained potato protease inhibitor II (PI2) that developed. And we demonstrated that this particular fiber formula was effective in preventing weight gain, increasing satiety signals, and reducing food intake in rats in a dosage‐dependent manner. Our study provides lines of evidence and would further bolster the use of this nutritious vegan meal in regulating satiety and food intake in clinics.

## Graphical abstract

A novel protein‐enriched fiber dietary supplement contained potato protease inhibitor II was developed. The satiety function evaluation of the fiber vegan product was carried out.

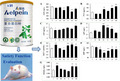

## 1 INTRODUCTION

For decades, obesity has been viewed as a grim public health issue global wide. Data from the Global Health Observatory (GHO) suggested that, in 2016, nearly 70% of people in the United States were suffered from either overweight or obese (Alvarado, 2019; Das & Khan, 2019; Middeldorp et al., 2019). Similarly, in China, the overweight rate had reached around 30% in 2016, which showed a roughly threefold increase of that rate in 1975 (Shi, Riley, Taylor, & Page, 2017; Zhang et al., 2016). In particularly, the prevalence rate of obesity in Chinese females is usually higher than that in Chinese males, which climbed from 0.7% in 1975 to 6.5% in 2016 (ninefold increase) (Zhang et al., 2017; Zhou, Zeng, Jin, & Cheng, 2017). Been obese would usually cause other metabolic and cardiovascular disorders, such as insulin resistance as well as abnormal blood lipid levels. Billions of dollars have been spend in dealing with obesity with respect to both keeping healthy life style as well as changing dietary habit.

Females usually care more about their body shape and health situation than males. Therefore, they are likely to be flexible in altering diet when aimed to lose weight. Compared to surgery and medicine application, calorie restriction or fasting is quicker and more feasible in losing weight. However, the reduction in the amount of food intake would possibly result in increased hungriness, malnutrition, and severe constipations due to fasting. All of which are unwanted and harmful outcomes. To avoid unnecessary hazards, safety should always be considered when looking for strategies that assist to reduce weight. Hence, many doctors have suggested weight control by adjusting eating behavior with special dietary supplements.

Growing evidence showed that dietary fiber with the unique physicochemical properties stimulate chewing and saliva secretion, which known to promote satiety signals (Rebello, O'Neil, & Greenway, 2016; Ye, Arumugam, Haugabrooks, Williamson, & Hendrich, 2015). Dietary fiber usually absorbs water and subsequently expands in the stomach to further increase the sense of satiety. The viscosity system is enhanced after fiber hydration, which slows stomach emptying and lengthens the sense of satiety. Epidemiological studies have showed that person who eats more fibers will tentatively be slimmer than those who eat fewer fibers (Tucker & Thomas, 2009). This finding further substantiates a negative correlation between eating dietary fibers and weight gain. Other than dietary fiber, potato extracts proteinase inhibitor II (PI2) enhances satiety by promoting endogenous release of cholecystokinin (CCK), a major satiety peptide produced by our body (Ku et al., 2016; Peters et al., 2011; Zhu, Lasrado, & Hu, 2017). Classic studies showed that CCK is a powerful endogenous peptide, which can prompt organs such as stomach and brain, to produce the sensation of satiety (de Graaf, Blom, Smeets, Stafleu, & Hendriks, 2004). In addition, studies have shown that high protein diet is also effective in weight control due to strong satiety when digesting proteins compared with other macronutrients (Deibert, Schmidt‐TrucksaessA, Frey, Landmann, & Berg, 2004; Mikkelsen, Toubro, & Astrup, 2000).

To date, satiety achieved by dietary fiber, PI2, or protein has only been studied separately. Little attention was given to the effect of the combination of these ingredients in weight control. The objective of this study was to evaluate the effect of satiety in a protein‐enriched dietary fiber formula (Vitamin World^®^ Vegan Meal) in rats, which is developed based on the satiety mechanism of oat fiber, potato extract PI2, and soybean protein isolate. Moreover, the formula was also compared with commercial competition of meal replacement powder to provide the evidence of the weight loss, food intake, and seven physiological indicators of satiety in rats, in order to develop the ideal product for weight control.

## 2 MATERIALS AND METHODS

### 2.1 Animals and diets

A total of 141 male Sprague Dawley (SD) rats with initial body weight of 180–200 g were provided by the Beijing Vital River Laboratory Animal Technology Co., Ltd. These rats were housed individually in standard cages that allowed recording of food intake. The cages were placed in a temperature‐ and humidity‐controlled room (25 ± 2℃, 55% ± 2% humidity) with a 12‐hr/12‐hr light/dark cycle (light off at 8:00 p.m.). Rats were given free access to water and food. The animals were acclimatized for one week with the control diet fed prior to the experiment and fasted 12 hr before gavage with free access to water. SD rats were used as they are reported to have a good consistency in satiety evaluation. All the experiment protocols were approved in accordance with the National Research Council's Guide for the Care and Use of Laboratory Animals.

### 2.2 Pilot study

In pilot study, 36 rats were randomly divided to six supplement treatment groups (6 rats per group). All diets were based on the normal chow (control diet). The blank control groups (B1, B2) were fed control diet with water. One pair of positive control groups P3 and P4 were fed the control diet with meal replacement powder (0.278 g/ml, Commercial competition). Another pair of positive control groups P5 and P6 were fed the control diet with potato extract (3.33 mg/ml, Slendesta^®^, Kemin Industries). Each rat in every group was given the test supplement 1.5 ml per day.

The pilot experiment was to investigate the time point (0.5, 1, 2, 4 hr) at which serum parameters changed significantly among Ghrelin, GLP‐1, GIP, CCK, PYY, LEP, and ADP after giving blank and positive control supplement, during which food intake was determined daily and body weights were measured.

On the first day, after intragastric administration, blood samples of B1, P3, and P5 were collected at 0.5 hr later, while blood samples of B2, P4, and P6 were collected at 1 hr later and the weight of the feeds in each group was measured. On the fourth day after 2 days of blank feeding, blood samples of B1, P3, and P5 were collected at 2 hr after intragastric administration, while blood samples of B2, P4, and P6 were collected 4 hr after intragastric administration and the weight of the feeds in each group was measured. The blood samples were stored in −80℃ for ELISA.

### 2.3 Formal experiment

The treatment supplements are Formula A (VW‐VM‐A), Formula A with low doses of PI2 (VW‐VM‐A/L), Formula A with high doses of PI2 (VW‐VM‐A/H), and Formula B (VW‐VM‐B), which were provided by Feihe Dairy Group. The ingredients of Formula A and B are soybean protein isolate, instant coconut powder, erythritol, soy protein powder, oat fiber, potato extract, mixed fruit powder, and siraitia grosvenorii powder, which contains 35% of protein and 14% of dietary fiber.

One positive control supplement is commercial competition of meal replacement powder which is a mature product on the market to enhance satiety at present. The potato extract was regarded as an another positive control supplement bought from Kemin Industries in the United States, a proved safe natural potato protein‐based dietary supplement ingredient that boosts satiety without any unpleasant side effects.

The formal experiment was to investigate the satiety of the VW‐VM Formula compared with commercial competition of meal replacement powder and potato extract PI2 from body weight, food intake, and serum parameters. The grouping and dosing of experiment are listed in Table 1. All of the 90 rats were acclimatized for one week with the control diet and weighed one day before the experiment. Each group began gavage at 7:00 a.m. every morning and collected the blood sample 1 hr later for 5 days. After 7 days of gavage weighed the rats separately and recorded the weight of the feed.

**TABLE 1:** Grouping and dosing of experimental animals

No.	Groups	Animals	Supplements	Doses
1	Blank control	15	Sterile distilled water	1.5 ml/d
2	Positive control 1	15	Potato protease inhibitor II (PI2)	25 mg/kg, 1.5 ml/d
3	Positive control 2	15	Commercial competition of meal replacement powder	2.08 g/kg, 1.5 ml/d
4	Treatment 1	15	Formula B (VW‐VM‐B)	2.08 g/kg, 1.5 ml/d
5	Treatment 2	15	Formula A(VW‐VM‐A)	2.08 g/kg, 1.5 ml/d
6	Treatment 3	15	Formula A with low doses of PI2 (VW‐VM‐A/L)	2.08 g/kg + 4.17 mg/kg 1.5ml/d
7	Treatment 4	15	Formula A with high doses of PI2 (VW‐VM‐A/H)	2.08 g/kg + 8.33 mg/kg 1.5 ml/d

### 2.4 ELISA analyses for satiety‐related indicators

Experiment instruments: Labsystems Multiskan MS, model352, Finland; Plate Washer: Thermo Labsystems, model AC8, Finland; Centrifuge: Model TG16W, China; Hydrostatic incubator: model TG16W, China; Electronic balance: Mettler XS105DU, Switzerland; Vortex generator: vortex‐genie2, United States.

Preparation of standard curve: 5 Eppendorf tubes were prepared and 150 μl diluted standard liquid were added in each tube (Tube 1–5). 150 μl of standard sample was added in Tube 1, and then, 150 μl of liquid from Tube 1was transferred into Tube 2. The rest was done in the same manner to achieve a serial dilution of standard sample with the concentration from high to low.

ELISA procedure: Each concentration of standard sample was measured twice, 50 μl/well, for parallel control. 50 μl of sample were loaded into each well included 40 μl of diluted sample liquid and 10 μl of sample. 50 μl of diluted standard liquid or sample liquid was added into blanks. Both standard and test sample adding were finished in 15 min and covered with plate sealing membrane and subsequently incubated in 37℃ for 30 min. 300 μL detergent was added and plate was placed onto Vortex generator for 5 s. Enzyme reagent (50 μL) was added for a 30‐min incubation for further color reaction. Finally, results were obtained by a plate reader. Then, the concentrations of Ghrelin, GLP‐1, GIP, CCK, PYY, LEP, and ADP in plasma were analyzed using an ultrasensitive rat enzyme‐linked immunosorbent assay kit (Biosource Inc.) according to the instructions.

### 2.5 Statistical analysis

Data were analyzed by Student's *t* test and one‐way ANOVA test by using StatView Statistics software program (Brainpower). Date were examined to ensure patterns of constant variance and a normal distribution. Results that were not fit to these conditions would be transformed to proper data using logarithms or square roots. The data were presented as means ± SEM, differences were considered significant if *p* < .05, and significant differences were represented as * for *p* < .05, ** for *p* < .01, and *** for *p* < .001.

## 3 RESULTS

### 3.1 VW‐VM‐A containing PI2 enhances satiety in rats by regulating serum PYY, Ghrelin, GLP‐1, CCK, and GIP

**FIGURE 1:**
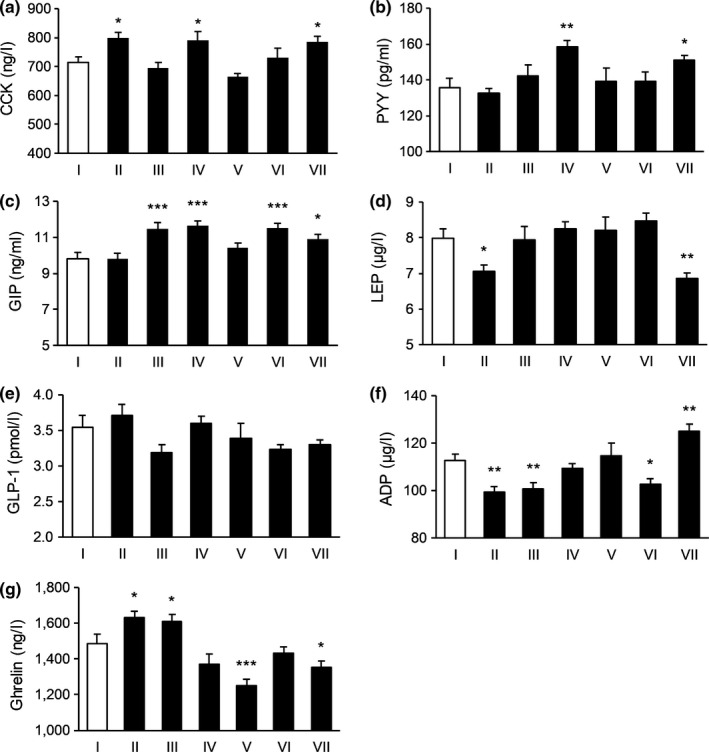
The effect of tested substances in regulating satiety signals in the first day. (a) The effect on the level of CCK; (b) the effect on the level of PYY; (c) the effect on the level of GIP; (d) the effect of the level of LEP; (e) the effect on the level of GLP‐1; (f) the effect on the level of ADP; and (g) the effect on the level of Ghrelin. *N* = 15 per each group. *p < .05, **p < .01, ***p < .001 versus control. I: Blank control, II: Potato extract PI2, III: Competing goods, IV: VW‐VM‐B, V: VW‐VM‐A, VI: VW‐VM‐A/L, and VII: VW‐VM‐A/H

**FIGURE 2:**
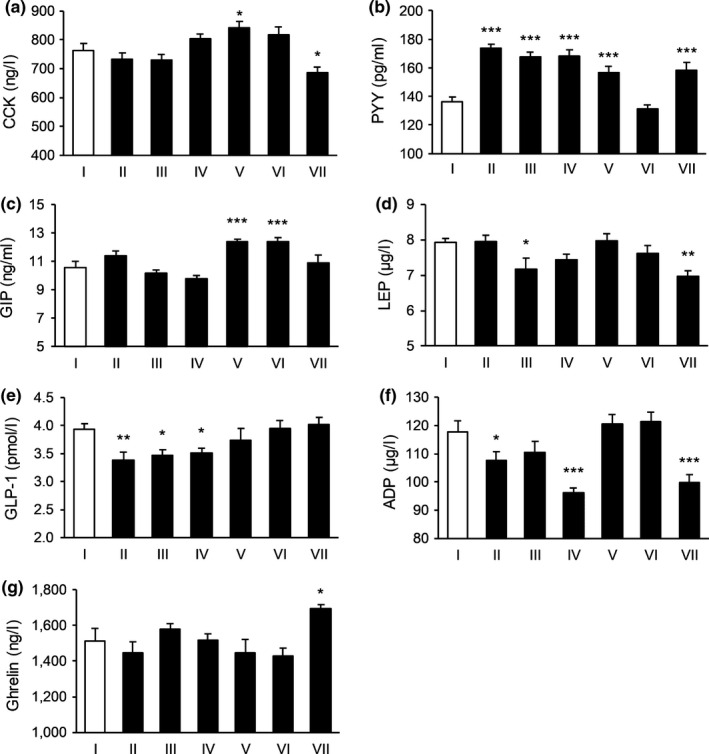
The effect of tested substances in regulating satiety signals in the second day. (a) The effect on the level of CCK; (b) the effect on the level of PYY; (c) the effect on the level of GIP; (d) the effect of the level of LEP; (e) the effect on the level of GLP‐1; (f) the effect on the level of ADP; and (g) the effect on the level of Ghrelin. *N* = 15 per each group. *p < .05, **p < .01, ***p < .001 versus control. I: Blank control, II: Potato extract PI2, III: Competing goods, IV: VW‐VM‐B, V: VW‐VM‐A, VI: VW‐VM‐A/L, and VII: VW‐VM‐A/H

**FIGURE 3:**
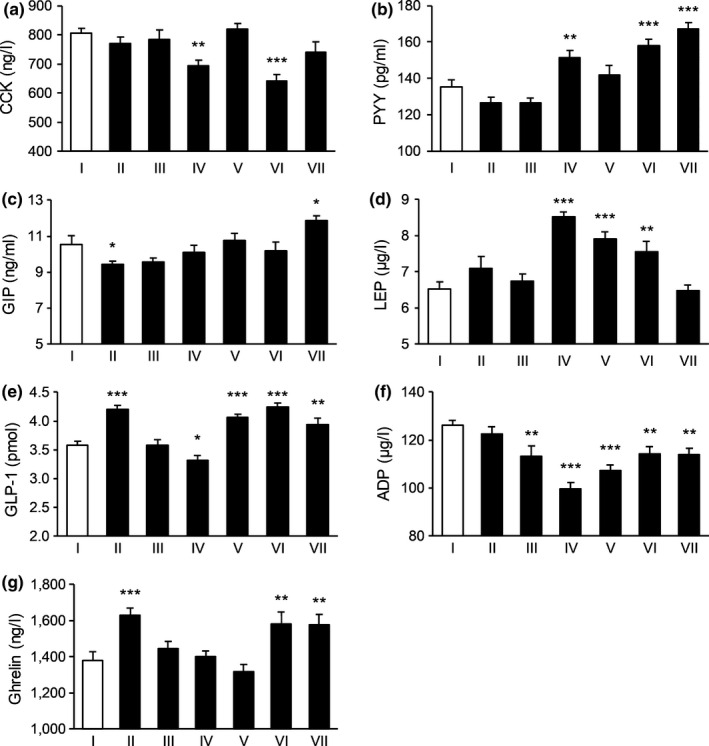
The effect of tested substances in regulating satiety signals in the third day. (a) The effect on the level of CCK; (b) the effect on the level of PYY; (c) the effect on the level of GIP; (d) the effect of the level of LEP; (e) the effect on the level of GLP‐1; (f) the effect on the level of ADP; and (g) the effect on the level of Ghrelin. *N* = 15 per each group. *p < .05, **p < .01, ***p < .001 versus control. I: Blank control, II: Potato extract PI2, III: Competing goods, IV: VW‐VM‐B, V: VW‐VM‐A, VI: VW‐VM‐A/L, and VII: VW‐VM‐A/H

**FIGURE 4:**
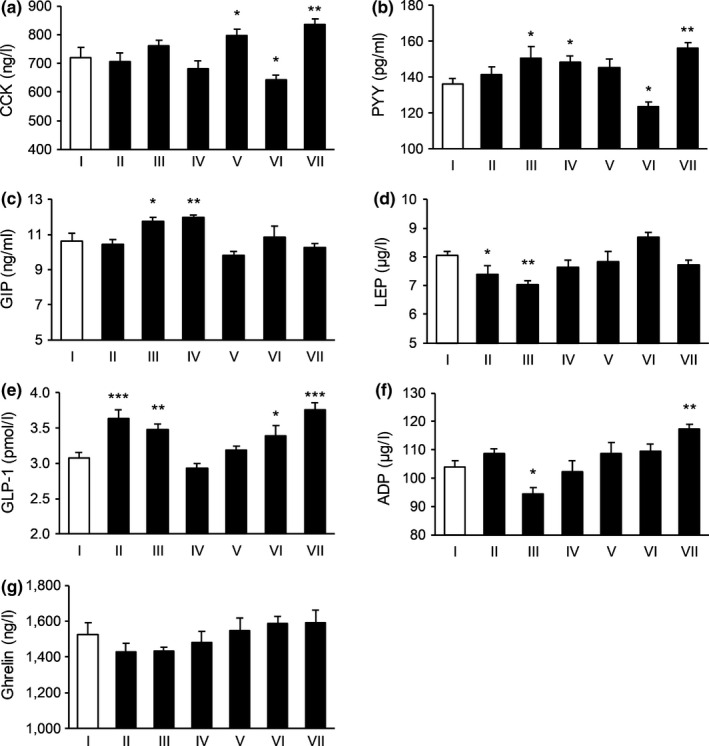
The effect of tested substances in regulating satiety signals in the fourth day. (a) The effect on the level of CCK; (b) the effect on the level of PYY; (c) the effect on the level of GIP; (d) the effect of the level of LEP; (e) the effect on the level of GLP‐1; (f) the effect on the level of ADP; and (g) the effect on the level of Ghrelin. *N* = 15 per each group. *p < .05,**p < .01,***p < .001 versus control. I: Blank control, II: Potato extract PI2, III: Competing goods, IV: VW‐VM‐B, V: VW‐VM‐A, VI: VW‐VM‐A/L, and VII: VW‐VM‐A/H

**FIGURE 5:**
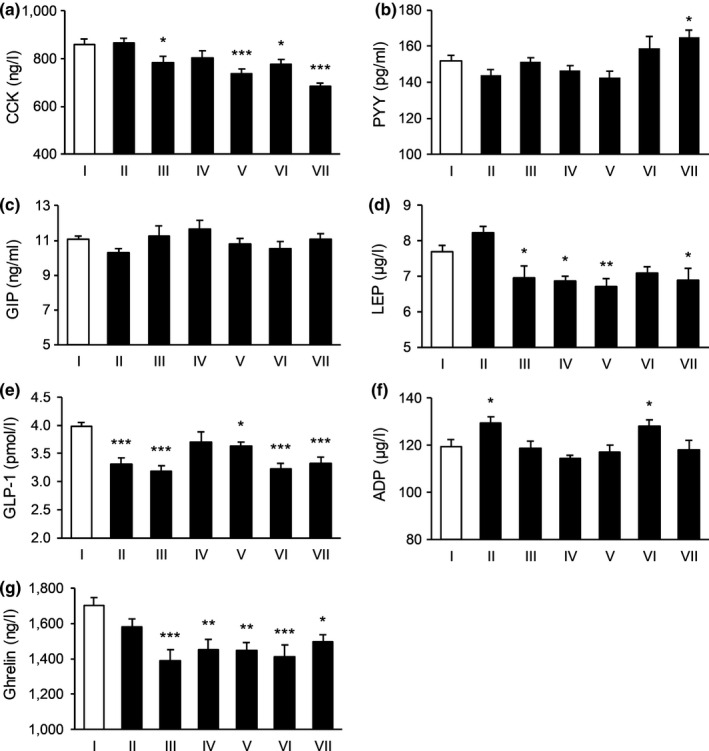
The effect of tested substances in regulating satiety signals in the fifth day. (a) The effect on the level of CCK; (b) the effect on the level of PYY; (c) the effect on the level of GIP; (d) the effect of the level of LEP; (e) the effect on the level of GLP‐1; (f) the effect on the level of ADP; and (g) the effect on the level of Ghrelin. *N* = 15 per each group. *p < .05, **p < .01, ***p < .001 versus control. I: Blank control, II: Potato extract PI2, III: Competing goods, IV: VW‐VM‐B, V: VW‐VM‐A, VI: VW‐VM‐A/L, and VII: VW‐VM‐A/H

The pilot study investigated the changes of CCK, PYY, GIP, LEP, GLP‐1, ADP, and Ghrelin levels in rat serum samples on 4 time points (0.5, 1, 2, and 4 hr) after gavage with sterile distilled water (blank control), potato extract PI2 (positive control 1), and commercial meal replacement powder (positive control 2). Our experiments were performed for five consecutive days, and data were averaged for all days for comparison but listed individually for each day in Table 2. We found that after 1 hr of gavage, all positive control groups could significantly enhance satiety revealed by the increased level of satiety peptides in serum, except LEP. The levels of PYY in serum after 2 hr of gavage and ADP in serum after 4 hr of gavage were also increased obviously. In addition, the serum level of CCK and ADP were elevated more by potato extract PI2 treatment than competing goods treatment, while the effect of the competing goods was more obvious on increasing GLP‐1 in serum. These results suggested that 1 hr would be an acceptable time point for us to determine the effect of different dietary supplements. Therefore, the sampling time was determined to be 1 hr after gavage in our formal experiments. Next, we determined the effect of Formulas A and B in regulating satiety‐related peptide in the serum. At least three independent trials on satiety were repeated (5 independent trials for 5 days) for statistic analysis. Remarkably, VW‐VM‐A/H formula increased the level of PYY (*n* = 5) (Figures 1, 2, 3, 4, 5).

**TABLE 2:** The effect of tested substances in regulating satiety signals (averaged from 5 days)

Group	Column 1	Column 2	Column 3	Column 4	Column 5	Column 6	Column 7
	Blank	PI2	Competing goods	VW‐VM‐B	VW‐VM‐A	VW‐VM‐A/L	VW‐VM‐A/H
CCK (ng/L) day 1–5							
Mean	771.9523	774.9978	751.176	754.5487	772.2814	722.5274	746.7457
SE	23.718	22.5874	23.6358	25.1532	18.4482	23.3104	21.4124
PYY (pg/ml) day 1–5							
Mean	139.0713	143.5221	147.6713	154.5596	145.1988	142.1931	159.6571
SE	3.6452	3.3282	4.202	3.5154	4.9662	4.115	3.6586
GIP (ng/ml) day 1–5							
Mean	10.52387	10.27528	10.84046	11.03107	10.82887	11.10166	11.00266
SE	0.3816	0.2596	0.3192	0.3172	0.2694	0.4034	0.3286
LEP (μg/ml) day 1–5							
Mean	7.638163	7.545495	7.168588	7.745666	7.729217	7.8856	6.986396
SE	0.1814	0.2324	0.269	0.174	0.2726	0.2126	0.184
GLP‐1 (pmol/L) day 1–5							
Mean	3.620514	3.647989	3.378046	3.411879	3.602283	3.605949	3.670383
SE	0.1	0.1186	0.0998	0.1006	0.1204	0.1066	0.1014
ADP (μg/L) day 1–5							
Mean	115.9952	113.593	107.6029	104.4001	113.7026	115.2303	114.8644
SE	2.7476	2.4736	3.2402	2.2718	3.6128	2.7424	2.7852
Ghrelin (ng/L) day 1–5							
Mean	1521.257	1544.229	1491.766	1444.322	1401.233	1488.352	1542.229
SE	56.362	46.7368	39.1518	49.4126	54.194	50.0184	46.1288

To be specific, on Day 1, Formula VW‐VM‐A/H and Formula B could significantly affect the levels of CCK, PYY, GIP, and Ghrelin, Formula VW‐VM‐A/L and positive control 2 strikingly affected the level of GIP, and Formula A decreased the level of Ghrelin and PI2 increased the level of CCK remarkably. The comparison result of influence degree for subject group targeted CCK was: PI2 > Formula B > VW‐VM‐A/H, and comparisons for the rest satiety indexes were displayed in Figure 1. On Day 2, Formula B increased the levels of CCK and PYY, while VW‐VM‐A/H and positive control 2 significantly increased the level of PYY. Formula A and VW‐VM‐A/L acted on CCK, GIP, and Ghrelin, while PI2 increased PYY, GIP, and Ghrelin remarkably. On Day 3, Formula B increased the levels of PYY and LEP, Formula A affected CCK, LEP, GLP‐1, and Ghrelin, VW‐VM‐A/L increased the levels of CCK, PYY, and GLP‐1, while VW‐VM‐A/H increased PYY, GIP, and GLP‐1 significantly. The influence degree of subject group acted on PYY was VW‐VM‐A/H > VW‐VM‐A/L > Formula B. On Day 4, Formula B increased the level of PYY, while VW‐VM‐A/H and positive control 2 increased CCK, PYY, and GLP‐1 strikingly. For the influence degree of CCK, VW‐VM‐A/H > Formula A > positive control 2, with respect to PYY, VW‐VM‐A/H > positive control 2 > Formula B. On the last day, all subject group could significantly affect the level of Ghrelin except for PI2. Besides, comparisons for the rest satiety indexes of Day 2–Day 5 were showed in Figures 2, 3, 4, 5.

In a summary, Formula VW‐VM‐A/L increased the level of GIP (*n* = 2) and CCK (*n* = 2) but showed little effect on GLP‐1 (*n* = 2). Whereas formula A has decreased Ghrelin level (*n* = 4). In addition, the effect of competing goods was reflected in PYY (*n* = 2) and GIP (*n* = 2). However, the effect of single potato extracts PI2 was not satisfactory, which mainly affected GLP‐1 (*n* = 2). These results indicated that the most effective formula among the testing supplements on increasing satiety was the Formula VW‐VM‐A/H. Moreover, Formula VW‐VM‐A/L and VW‐VM‐A/H were found to be the most effective dietary supplement in causing satiety. Importantly, all tested groups (Formula A, VW‐VM‐A/L, and VW‐VM‐A/H) were shown to be effective on increasing satiety.

### 3.2 Dietary supplements did not affect body weight gain

After evaluating serum markers for satiety, we looked into the body weight gain in rats treated with different dietary supplements. We found a reduced body weight gain in all Formula A containing groups when 7 days of continuous gavage were introduced (see Table 3). These results suggested that Formulas A is capable of preventing excessive body weight gain in adult rats provided with normal chow.

**TABLE 3:** Effect of the test supplements on rats’ body weight

Groups	Blank Control	PI2	Competing goods	VW‐VM‐A	VW‐VM‐A/L	VW‐VM‐A/H
Weight gain (g, mean ± SE)	33.81 ± 8.50	31.92 ± 6.19	32.70 ± 5.24	31.22 ± 7.3^*^	30.78 ± 8.1^*^	31.45 ± 6.5^*^

### 3.3 The effect on food intake

The total food intake of every cage was weighed continuously for 5 days after gavage at 0.5, 1, and 6 hr every day. Average daily food intake throughout the experiment was affected by the test supplements.

**FIGURE 6:**
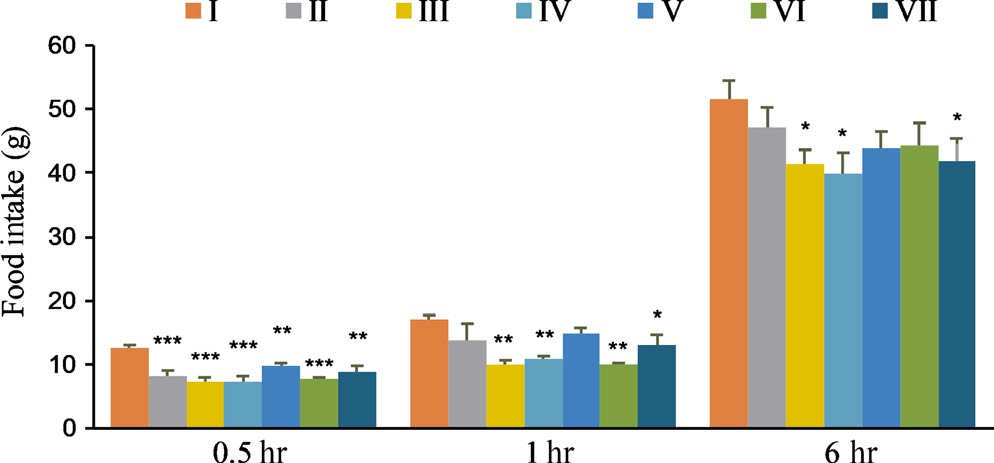
Effect of the Test Supplements on Rats’ food intake. *p < .05, **p < .01, ***p < .001 versus control. I: Blank control, II: Potato extract PI2, III: Competing goods, IV: VW‐VM‐B, V: VW‐VM‐A, VI: VW‐VM‐A/L, and VII: VW‐VM‐A/H

The result showed the blank control group had the highest food intake (Table 4). Food intake of positive control groups and Formula VW‐VM groups were decreased compared with the control group at different time points (0.5, 1, and 6 hr) (Figure 6). These results suggested that Formula VW‐VM‐A/H and VW‐VM‐B were relatively most effective in reducing food intake.

**TABLE 4:** Effect of the test supplements on rats’ food intake (averaged from five days)

Groups	Column 1	Column 2	Column 3	Column 4	Column 5	Column 6	Column 7
	Blank	PI2	Competing goods	VW‐VM‐B	VW‐VM‐A	VW‐VM‐A/L	VW‐VM‐A/H
0.5‐hr food intake(g)							
Mean	12.527	8.163	7.400	7.397	9.733	7.683	8.847
SE	0.575	0.861	0.475	0.729	0.366	0.262	0.978
1‐hr food intake (g)							
Mean	17.123	13.683	10.083	10.850	14.833	9.957	13.100
SE	0.703	2.696	0.643	0.52	0.885	0.247	1.537
6‐hr food intake (g)							
Mean	51.673	47.133	41.417	39.970	43.953	44.213	41.797
SE	2.9	3.258	2.128	3.29	2.676	3.595	3.667

## 4 CONCLUSIONS

In this study, we systematically evaluated the "Vitamin World^®^ Vegan Meal" Formula of *Feihe* containing dietary supplement's function in regulating satiety, body weight gain and food intake using rats as a model. By measuring variety of biological markers related with satiety, we reveled a remarkable role of Formula VW‐VM‐A/L and VW‐VM‐A/H were capable of promoting satiety by increasing PYY, CCK, GIP, and ADP as well as decreasing LEP and Ghrelin. Interestingly, we noted that high dosage of Formula VW‐VM‐A/H exhibited stronger effect on increasing the level of PYY, CCK, and ADP, and decreasing LEP and Ghrelin than low dosage Formula A. We reason that Formula A contained PI2 may promote satiety in a dosage‐dependent manner. However, GIP was not further increased upon high dosage treatment compare with low dosage Formula A treatment. This particular phenotype could be due to a strong effect on other satiety‐related factors which might in turn suppress the disproportionately enhanced level of GIP. In addition, we observed that Formula VW‐VM‐A/H and VW‐VM‐B successfully attenuated excessive body weight gain in rats and inhibited food intake for days. These results were consistent and provide numbers of evident to support our hypothesis that Formula VW‐VM containing dietary supplement was effective in suppressing body weight gain and food intake chronically. We therefore view Formula VW‐VM‐A/H as a competent dietary supplement for regulating daily food consumption in health subjects which could potentially be used as a tool for bodyweight control. Rats given Formula VW‐VM‐A/H gavage reduced the food intake, promoted satiety, and reduced body weight gain.

## CONFLICT OF INTEREST

The authors declare that they have no conflict of interest to this research.

## INFORMED CONSENT

Written informed consent was obtained from all study participants.
